# Transcriptome analyses of sex differential gene expression in brains of rare minnow (*Gobiocypris rarus*) and effects of tributyltin exposure

**DOI:** 10.1016/j.dib.2018.03.119

**Published:** 2018-03-29

**Authors:** Ji-liang Zhang, Chun-nuan Zhang, Min Liu, Ming-zhen Fan, Mao-xian Huang

**Affiliations:** Laboratory of Aquatic Environment and Animal Safety, College of Animal Science and Technology, Henan University of Science and Technology, 263 Kaiyuan Road, Luoyang 471023, Henan, China

## Abstract

RNA-sequencing was used to identify sex-biased gene expression in brains of rare minnow (*Gobiocypris rarus*) by comparing transcriptomic profiles between females and males. Furthermore, transcriptomic responses to 10 ng/L tributyltin (TBT) in both male and female brains were also investigated to understand whether TBT affects the identified sex-biased genes. Differentially expressed genes (DEGs) were identified using the IDEG6 web tool. In this article, we presented male- and female-biased DEGs, and up-regulated and down-regulated DEGs after TBT exposure. The raw reads data supporting the present analyses has been deposited in NCBI Sequence Read Archive (SRA, http://www.ncbi.nlm.nih.gov/Traces/sra) with accession number PRJNA376634. The data presented in this article are related to the research article entitled “Transcriptomic analyses of sexual dimorphism of rare minnow (*G. rarus*) brains and effects of tributyltin exposure” (doi: 10.1016/j.ecoenv.2018.02.049).

**Specifications table**

**Table:** 

Subject area	*Biology*
More specific subject area	*Aquatic toxicology*
Type of data	*Excel files*
How data was acquired	*High-throughput RNA-sequencing*
Data format	*Filtered and analyzed with statistical tests*
Experimental factors	*The adult rare minnows (Gobiocypris rarus) were exposed to 10 ng/L of tributyltin or only equal volume of the solvent (1 μL/L 95% ethanol) as control, after 60 d the brains were sampled and frozen*
Experimental features	*RNA was isolated from the brains and sequencing was performed on the Illumina HiSeq. 2500 platform. Differential gene expression analysis was performed by the IDEG6 web tool.*
Data source location	*N/A*
Data accessibility	*Analyzed datasets are contained within this article. Raw data was deposited in the NCBI database Sequence Read Archive under accession number PRJNA376634.*

**Value of the data**

• The data show brain differentially expressed genes between male and female rare minnow, which could provide key information on the molecular basis of brain sexual dimorphism in fish.
• The sexually dimorphic gene could provide valuable resources for investigating molecular mechanisms of sexual plasticity in response to environmental influences.
• Analysis of the brain differentially expressed genes after tributyltin exposure would help to understand the molecular mechanisms of tributyltin toxicity.

## 1 Data

Brain transcriptome sequencing from control and tributyltin (TBT) exposed rare minnows was performed. The dataset of this article comprises 5 data files that generated from the differential gene expression analysis. The significantly differentially expressed genes (DEGs) in comparisons of Female vs. Male are presented in File 1. The DEGs in comparisons of Control vs. TBT in female fish are presented in File 2. The DEGs in comparisons of Control vs. TBT in male fish are presented in File 3. Among the sex-biased DEGs, the up- or down-regulated DEGs in female fish after TBT exposure are presented in File 4. The up- or down-regulated sex-biased DEGs in male fish after TBT exposure are presented in File 5.

## 2 Experimental design, materials, and methods

### 2.1 Chemicals and experimental species

The adult rare minnows were sex-separated and randomly assigned into glass tanks and exposed to TBT at nominal concentrations of 10 ng/L (84 pmol/L) or only equal volume of the solvent (1 μL/L 95% ethanol) as control for 60 d. At the end of the exposure, the brains were dissected out and used for Illumina sequencing.

### 2.2 Illumina sequencing

Three samples from the control or TBT exposure group were used for RNA isolation, as described in [1]. Sequencing was performed on Illumina HiSeq. 2500. The mRNA sequencing library was constructed as described previously [2]. High-quality reads (quality percentage value ≥ Q30%) were *de novo* assembled using the Trinity program [3]. Putative functions of unigenes were identified using BLAST (E-value ≤10^−5^) against public database including National Center for Biotechnology Information non-redundant protein database, Swiss-prot database, Clusters of Orthologous Groups, Gene Ontology, and Kyoto Encyclopedia of Genes and Genomes databases.

### 2.3 Identification of DEGs

The gene expression was measured as reads per kilo base of transcripts per million (RPKM) according to the method described in Mortazavi et al. [4]. Detection of DEGs was performed by the IDEG6 web tool [5], and the results of all statistical tests were corrected by multiple testing with the Benjamini-Hochberg false discovery rate control (FDR < 0.01). Genes were defined as differentially expressed by parameters of FDR < 0.01 and the absolute value of the log_2_ ratio ≥ 1.
